# Correction to: A Systematic Literature Review of Information Sources for Threat Modeling in the Power Systems Domain

**DOI:** 10.1007/978-3-030-58295-1_8

**Published:** 2020-08-26

**Authors:** Engla Ling, Robert Lagerström, Mathias Ekstedt

**Affiliations:** 8 Department of Computer Science, University of Bristol, Bristol, UK; 9 Department of Computer Science, City, University of London, London, UK; Division of Network and Systems Engineering, KTH Royal Institute of Technology, Stockholm, Sweden

## Correction to: Chapter “A Systematic Literature Review of Information Sources for Threat Modeling in the Power Systems Domain” in: A. Rashid and P. Popov (Eds.): *Critical Information Infrastructures Security*, LNCS 12332, 10.1007/978-3-030-58295-1_4

Chapter 4, “A Systematic Literature Review of Information Sources for Threat Modeling in the Power Systems Domain” was previously published non-open access. It has now been changed to open access under a CC BY 4.0 license and the copyright holder updated to ‘The Author(s)’. The book has also been updated with this change.

